# Anatomy of open access publishing: a study of longitudinal development and internal structure

**DOI:** 10.1186/1741-7015-10-124

**Published:** 2012-10-22

**Authors:** Mikael Laakso, Bo-Christer Björk

**Affiliations:** 1 Hanken School of Economics, Helsinki, Finland

**Keywords:** Open access, scientific publishing

## Abstract

**Background:**

Open access (OA) is a revolutionary way of providing access to the scholarly journal literature made possible by the Internet. The primary aim of this study was to measure the volume of scientific articles published in full immediate OA journals from 2000 to 2011, while observing longitudinal internal shifts in the structure of OA publishing concerning revenue models, publisher types and relative distribution among scientific disciplines. The secondary aim was to measure the share of OA articles of all journal articles, including articles made OA by publishers with a delay and individual author-paid OA articles in subscription journals (hybrid OA), as these subsets of OA publishing have mostly been ignored in previous studies.

**Methods:**

Stratified random sampling of journals in the Directory of Open Access Journals (n = 787) was performed. The annual publication volumes spanning 2000 to 2011 were retrieved from major publication indexes and through manual data collection.

**Results:**

An estimated 340,000 articles were published by 6,713 full immediate OA journals during 2011. OA journals requiring article-processing charges have become increasingly common, publishing 166,700 articles in 2011 (49% of all OA articles). This growth is related to the growth of commercial publishers, who, despite only a marginal presence a decade ago, have grown to become key actors on the OA scene, responsible for 120,000 of the articles published in 2011. Publication volume has grown within all major scientific disciplines, however, biomedicine has seen a particularly rapid 16-fold growth between 2000 (7,400 articles) and 2011 (120,900 articles). Over the past decade, OA journal publishing has steadily increased its relative share of all scholarly journal articles by about 1% annually. Approximately 17% of the 1.66 million articles published during 2011 and indexed in the most comprehensive article-level index of scholarly articles (Scopus) are available OA through journal publishers, most articles immediately (12%) but some within 12 months of publication (5%).

**Conclusions:**

OA journal publishing is disrupting the dominant subscription-based model of scientific publishing, having rapidly grown in relative annual share of published journal articles during the last decade.

## Background

Open access (OA) has expanded the possibilities for disseminating one's own research and accessing that of others [1,2]. OA, in the context of scholarly publishing, is a term widely used to refer to unrestricted online access to articles published in scholarly journals. There are two distinct ways for scholarly articles to become available OA, either directly provided by the journal publisher (gold OA), or indirectly by being uploaded and made freely available somewhere else on the Web (green OA). Both options increase the potential readership of any article to over a billion individuals with Internet access and indirectly speed up the spread of new research ideas. While the majority of OA journals do not charge authors anything for the services provided, a growing minority of professionally operating journals charge authors fees ranging from 20 to 3800 USD, with an estimated average of 900 USD [3].

OA is closely related to developments in other media content delivery businesses, and its ethos is well aligned with the fundamental openness principle of science itself as well as the ideologies behind Wikipedia and open source software. However, what makes scientific publishing distinct is the influence journal prestige and rankings have on journal selection for authors submitting article manuscripts [4]. There are also vested interests to preserve the status quo of the current subscription market among stakeholders, with dominant publishers seeing OA as a potential threat to the bottom-line. Friction caused by these and other factors can be argued to slow down the process of OA adoption because journals are not direct substitutes for each other and subscription-based journal copyright agreements can prohibit parallel distribution of published content. However, following in the footsteps of the National Institutes of Health in the US, public research funders in the UK have recently launched strategies to increase OA to publicly funded research [5]. While the ultimate goal of increasing access to publicly funded research is known and widely accepted it is difficult to reach compromises that balance the long- and short-term interests of the stakeholders involved [6].

Important changes in policy facilitating growth of OA happen on many levels, influencing research publishing both upstream and downstream. The examples from the public funders in the US and UK are merely the most ambitious movements so far: public and private research funders large and small, universities, publishers and research institutes all contribute to forming the evolving OA landscape. The problem that has persisted with OA since the start is the lack of readily available data for how this particular subset of journal publishing is developing over time, an aspect which is described in closer detail in the Methods section. Policymakers should have an interest in knowing how common OA is today, how fast the share of OA has increased and what proportion of journal articles are currently OA? The purpose of this study is to provide answers to these types of questions.

### Aim of the study

This study focuses on providing measurement of the longitudinal development gold OA publication volume for the years 2000 to 2011 as a whole and by subtype: full immediate journal OA, delayed OA and hybrid OA. As will be described in more detail further on, earlier studies have mostly ignored the subset of delayed OA journals. This is partly because there is no comprehensive index of such journals similar to the service the Directory of Open Access Journals (DOAJ) provides for immediate OA journals, and partly because of the divisive acceptance of delayed OA as a valid form of OA. However, the subset of delayed OA journals is both substantial in volume and is populated with many high-quality journals; five of the 10 most-cited journals within Thomson Reuters Web of Knowledge in the period from 1999 to 2009 are currently delayed OA while the others are subscription-access only [7]. Hybrid OA is the term commonly used for describing individual articles being provided openly within subscription-only journals through an optional author payment; it is only recently that this type of OA has been properly studied [8].

The chosen research aim is related to some existing areas of OA research that warrant mention to clarify the specific contribution of this study. Green OA is not part of the scope of this study as that is a wholly different research problem and one that requires its own set of methods, as different versions of articles are scattered around on the Web. Furthermore, this study does not extensively discuss or evaluate the pros or cons of OA, since there is already a well-developed body of literature focusing on issues such as relationships between OA and readership, citation or impact [9-12]. In summary, the aim is to provide comprehensive and up-to-date quantitative measurement of gold OA journals and articles. The results and data of this study can then potentially act as a foundation for more targeted research enquiries.

### Previous studies

Researchers have applied different methods to cope with the lack of readily available quantitative data to study the OA phenomenon, ranging from labor-intensive manual article-counting [13-15] to automated Web-crawling [16,17]. What is known about the early years of OA, both gold and green, is mostly through a series of independent studies providing snapshots for individual years based on sampling various publication indexes. The fact that studies have been based around OA prevalence within different publication indexes and the diverse adopted sampling methods makes comparisons or composition of longitudinal development inexact. Nevertheless, these are the best figures currently available. The earliest comprehensive study suggests the 2003 share for gold OA to have been 2.9% for articles included in the Thomson Reuters Web of Knowledge [18]. The next study was performed for the 2006 publication volume based on data from UlrichsWeb [19] and the DOAJ [20], where a gold OA share of 8.1% and a green OA share of 11.3% resulted in a combined OA share of 19.4% [14]. For 2008 articles, the Thomson Reuters Web of Knowledge gold OA share was measured to be 6.6% and green OA 14%, resulting in a figure of total OA of 20.6% [21]. Also for 2008, a large-scale study based on English-language journals listed in the DOAJ calculated that 120,000 articles were published OA either through full immediate OA journals or as individual hybrid OA articles [22]. The first comprehensive longitudinal study on the volume of articles published by full immediate OA journals in the DOAJ resulted in an average annual year-on-year growth rate of 30% from 2000 to 2009, with some 191,000 articles published during 2009 [13]. Another longitudinal study, including both gold and green OA, produced a total OA share of 23.1% for Thomson Reuters Web of Knowledge indexed articles published during 2010 [16]. Outside of this 2010 study of Thomson Reuters Web of Knowledge, there are no comprehensive measurements for OA volume since 2009. This study is designed to provide a longitudinal study implementing a well-documented and easily replicable methodology, producing results applicable to multiple publication indexes, producing results that are easy to follow-up and compare with future measurements.

## Methods

### Sampling

**Figure 1:**
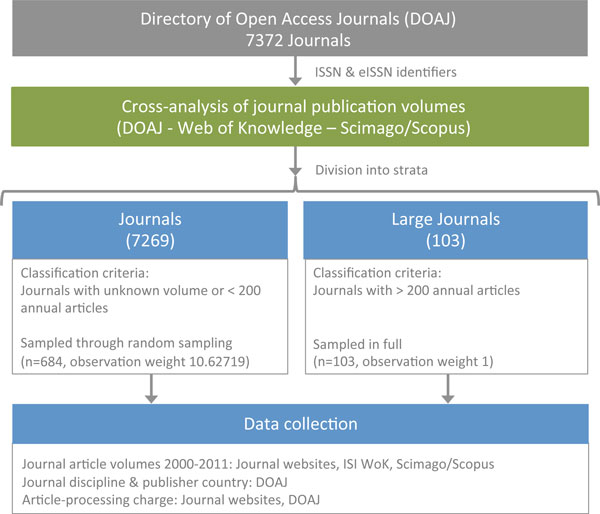
**Visualization of the sampling**.

The study is founded on the assumption that the full population of OA journals is listed in the DOAJ. There are OA journals not indexed in this database, but systematically identifying them is not feasible. Because the majority of the 7,372 journals listed in the DOAJ on 1 January 2012 were not included in any indexing service that would reliably keep track of their article output, nor the exact year previously subscription-based journals have converted to OA, gathering data is largely a manual task and one of the major practical challenges for the execution of studies of this type. To strike a balance between feasibility and reliability, stratified random sampling with unequal probabilities was utilized, a sampling method that has proven suitable for similar studies in the past [13]. An argument for adopting this approach in favor of fully random sampling is that the population of OA journals is highly heterogeneous, where a small number of titles output a large proportion of the total article volume [22]. The fact that large journals can be identified with a high degree of certainty through various indexing services also means that reliable, readily available article count information can be used for journals responsible for a major part of the total OA output. A visualization of the sampling is provided in Figure 1A cross-analysis of data available from SCImago [23], Thomson Reuters Web of Knowledge [24] and the DOAJ identified 103 OA journals that had published over 200 articles annually during 2009, 2010 or 2011; these were included in the large journal stratum. The rest of the 7,269 DOAJ journals were represented by a second stratum with a sample of 684 journals selected at random among them, each given an observation weight of 10.62719 (684 × 10.62719 = 7269). The stratum of large journals was only applied an observation weight of 1 since the population of that stratum is exhaustively sampled.

### Data collection

Through a previous study using identical sampling and data collection methodology [13], data for 565 journals spanning publication volumes for 2000 to 2009 could be re-used, with only the need to gather publication volumes for two additional years. Since the existing data material lacked coverage for journals added to the DOAJ during 2010 and 2011, an additional randomly selected sample was drawn out of the journals added within the two missing years adhering to the same sampling probability as the pre-existing sample (0.1011), with 222 new journals added to the existing sample of 565 journals.

Where journal publication volumes could be retrieved from either SCImago or Thomson Reuters Web of Knowledge, such data was used. For the majority of journals, the individual journal websites were visited and the annual entries collected manually. It is worthwhile to note that journals often include editorials, news, book reviews, obituaries and other non-research content. Such material was excluded from all measurements in this study. To provide an accurate representation of retrospective OA volume, articles were not collected for subscription-only journals prior to publishing OA. Determining when a journal has initiated OA publishing often requires manual investigation as the information is not always made explicit on the webpages, and the data concerning this is often incorrect in the journal metadata available in the DOAJ. To support the analysis of the sampled journals, additional data from Scopus [25] and Thomson Reuters Web of Knowledge was utilized in addition to the data that is already available through the DOAJ.

## Results

**Figure 2:**
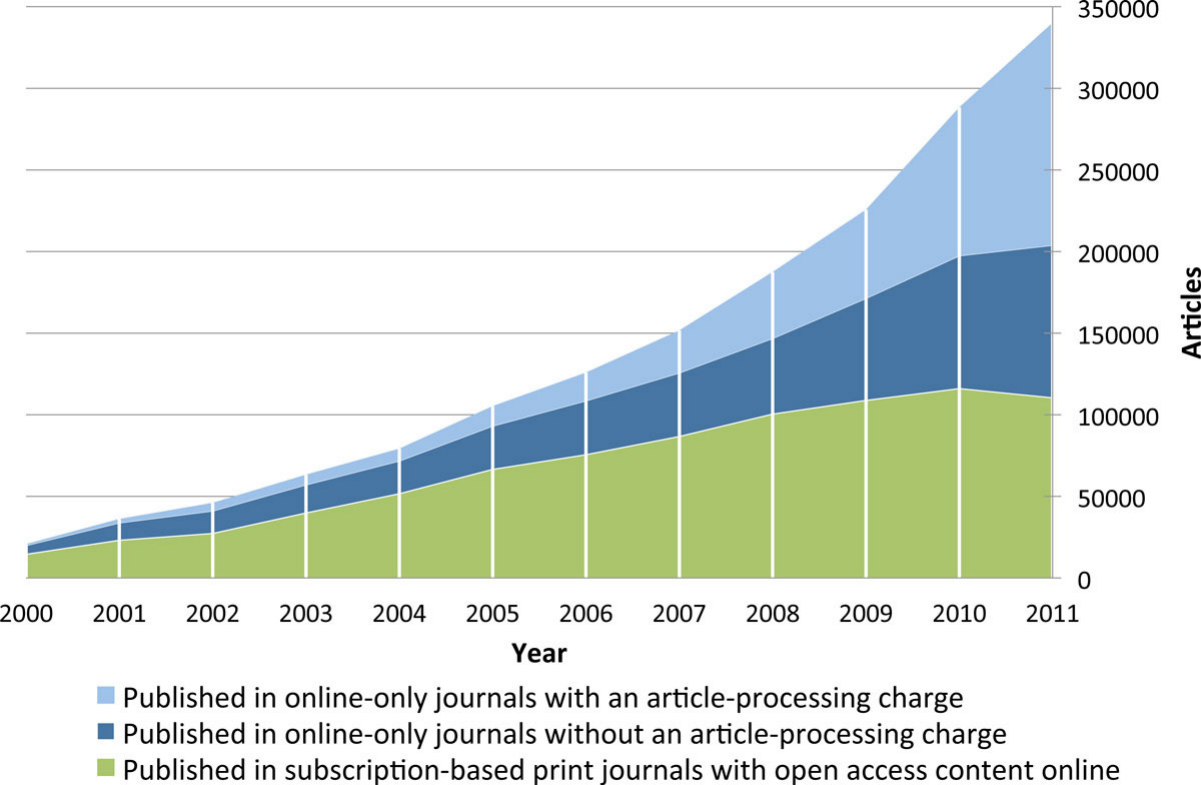
**Annual volumes of articles in full immediate open access journals, split by type of open access journal**.

The longitudinal development of full immediate OA article volume spanning 2000 to 2011 is presented visually in Figure 2 and numerically in Table 1, where a breakdown of the total volume is provided for articles split into three different categories: online-only journals that require an article-processing charge, online-only journals that do not require an article-processing charge, and journals that still output print versions for subscribers but have all articles available OA online. It is important to point out that journals still producing a print version might also require an article-processing charge in addition to having income from subscriptions. However, such differentiation is not provided here due to the relative rarity of such journals as well as a desire to focus on these three mutually exclusive business models specifically.

**Table 1:** Estimated annual article and journal counts in full immediate open access journals

	Year	2000	2001	2002	2003	2004	2005	2006	2007	2008	2009	2010	2011
**Online-only OA journals (APC)**	**Articles**	795	2332	4,936	6,247	7,532	12,143	17,256	25,949	40,689	54,296	90,932	136,264
	**Journals**	53	120	167	189	256	344	425	630	950	1,239	1,494	1,824
													
**Online-only OA journals (no APC) **	**Articles**	5,445	10,690	13,844	17,238	20,106	26,626	33,067	38,991	46,362	62,521	81,421	93,513
	**Journals**	334	484	613	804	1,006	1,272	1,538	1,793	2,048	2,399	2,548	2,495
													
**Subscription-based print journals with OA content online**	**Articles**	14,461	23,095	27,234	39,814	51,614	66,494	75,486	86,691	100,393	108,793	116,003	110,353
	**Journals**	357	550	630	847	1,106	1,375	1,539	1,819	2,011	2,149	2,170	2,395
													
**All OA journals**	**Articles**	20,702	36,117	46,013	63,299	79,253	105,262	125,809	151,630	187,444	225,610	288,357	340,130
	**Journals**	744	1,154	1,410	1,841	2,368	2,991	3,502	4,243	5,010	5,788	6,213	6,713

APC: article-processing charge; OA: open access.

Overall there has been growth in the annual output among all three categories since the year 2000, going from a total volume of 20,700 articles in 2000 to 340,000 in 2011. Not depicted in Figure 2 but provided in Table 1 is the number of active OA journals for each respective year (journals with at least one article published during the respective year), which has increased from 744 journals in the year 2000 to 6,713 in 2011. The average number of articles per journal has also seen a constant increase, with an average of 26 articles per journal in 2000, 33 in 2005, and 51 for 2011. However, a reminder about the skewed nature of article distribution among journals is relevant here. There is a handful of journals publishing more than 1,000 articles per year and thousands of journals publishing only a few articles annually.

Inspecting the internal structure of the total article mass reveals some major shifts that have happened over the course of a decade. Journals that also publish a parallel print version, which are often old, established journals that decided to make the online version free when they started putting their content on the Web, provided the majority of the OA content up until the year 2008 where, for the first time, online-only journals took the lead in terms of output volume. Since 2008, the online-only journals have sustained a much stronger growth while the OA output provided by journals outputting a print version has plateaued to annual volumes between 100,000 and 110,000 articles. The latter group includes a lot of society journals registered with dedicated portals like SciELO [26], Redalyc [27] and J-Stage [28] providing the technical platform for electronic publishing. Journals with author-processing charges have seen breakout growth during the last three years, going from 80,700 articles in 2009 to 166,700 articles in 2011.

Cross-analysis of the sample with the titles listed in Thomson Reuters Web of Knowledge index and Elsevier's Scopus index was performed, only including the titles present in the respective index to calculate the share of OA articles of all peer-reviewed articles. Table 2 provides the main results of this analysis, presented as longitudinal breakdowns of publisher-provided OA in the two indexing services. Nearly half of all full immediate OA articles published during 2011 were outside of Scopus and two thirds outside of Thomson Reuters Web of Knowledge, meaning that a large portion of article OA article volume lacks coverage in major publication indexes. This issue highlights the importance of using manual data collection methods in OA studies because data available from indexes only provide part of the total picture. In addition to the results concerning full immediate OA journals, Table 2 also contains volume data for two other types of publisher-provided OA in each respective index: delayed OA and hybrid OA.

**Table 2:** Proportion of publisher-provided (gold) open access in major indexes

Articles indexed in Scopus	2008	2009	2010	2011
Total^a^	1,469,286	1,550,413	1,588,636	1,658,643
In full immediate OA journals^b ^	118,751	133,817	163,670	181,706
**Share of articles published in full immediate OA journals **	**8.1%**	**8.6%**	**10.3%**	**11%**
Hybrid OA^c^	4,718	8,095	10,135	12,089
Delayed OA^d^	78,054	82,271	81,404	85,714
**Total share OA**	**13.7%**	**14.5%**	**16.1%**	**16.9%**

**Articles indexed in Web of Knowledge**	**2008**	**2009**	**2010**	**2011**

Total^e^	1,154,803	1,203,692	1,235,202	1,294,051
In full immediate OA journals^b^	76,537	85,852	103,514	116,192
**Share of articles published in full immediate OA journals **	**6.6%**	**7.1%**	**8.4%**	**9.0%**
Hybrid OA^c^	3774	6476	8108	9671
Delayed OA^d^	76,076	80,338	79,058	83,420
**Total share OA**	**13.5%**	**14.3%**	**15.4%**	**16.2%**

^a^All articles + reviews, retrieved 22 May 2012 through scopus.com; ^b^results of this study; ^c^results of previous research [8]; ^d^results of an ongoing study by the authors, based on absolute volume calculations for articles in 462 unique journals available through HighWire Press, PubMed Central, Elsevier and so on with an open access delay of 12 months or less; ^e^all articles + reviews included in the Science Citation Index Expanded, Social Sciences Citation Index, Arts & Humanities Citation Index, retrieved 22 May 2012 through apps.webofknowledge.com. OA: open access.

Of the 1.66 million articles indexed by Scopus in 2011, 11% were published in full immediate OA journals, 0.7% as hybrid OA and 5.2% in journals that have a maximum OA delay of 12 months. Together, these account for almost 17% of the total article volume in the whole index. The figures for articles indexed by Thomson Reuters Web of Knowledge are comparable to those of Scopus, with a total publisher-provided OA rate of 16.2% for 2011. Of the 1.29 million articles indexed by Thomson Reuters Web of Knowledge, 7.9% are available in full immediate OA journals, 0.7% as hybrid OA and 6.4% in journals that have a maximum OA delay of 12 months. Overall the results suggest that there has been an increase of about one percentage point annually in relative OA volume in both Scopus and Thomson Reuters Web of Knowledge during 2008 to 2011.

**Figure 3:**
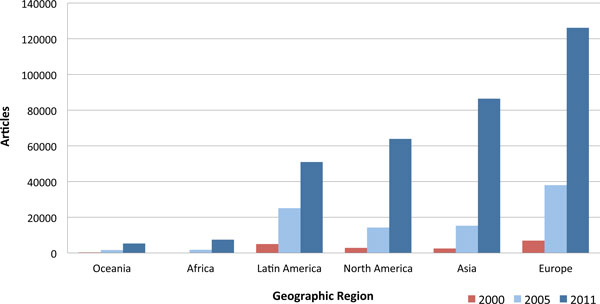
**Open access publisher output across geographic regions**.

Figure 3 presents the longitudinal development of OA publisher output as measured by the number of articles output by publishers based in different regions of the world. This figure, and all that follow, only includes full immediate OA journals, excluding delayed and hybrid OA. Prior to interpretation it needs to be noted that this is a publisher-centric analysis. In some cases, the publisher is not registered within the same country, or even region of the world, as the journal. The results suggest that Latin American countries were early to have substantial OA output, possibly due to the early availability of the SciELO portal. However, the region has not increased its output at a similar rate as North America, Asia or Europe, who have multiplied their outputs between 2005 and 2011.

**Figure 4:**
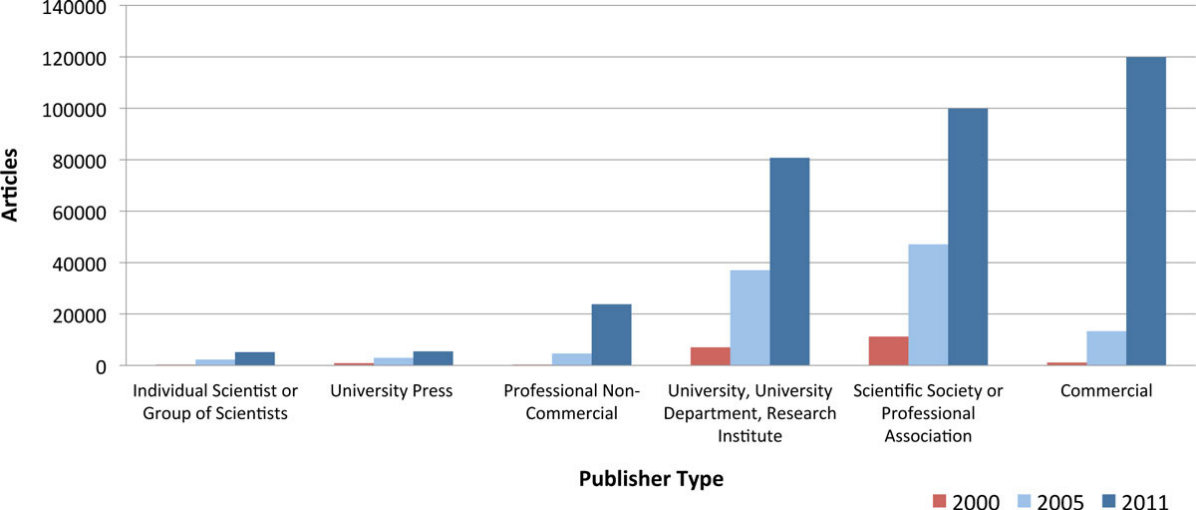
**Open access publisher type analysis**.

Figure 4 presents the total OA article volume for 2000, 2005 and 2011 split according to publisher type. The analysis shows that the early years of OA publishing were largely driven by scientific societies, professional associations, universities and their departments as well as individual scientists. Scientific societies and universities have maintained strong growth throughout the decade, while scientist-driven publication has been overshadowed by the article volume produced by the more formally organized publisher types. The most dramatic development since 2005 is the rapid increase in articles published by commercial publishers, jumping from 13,400 articles in 2005 to 119,900 in 2011, resulting in commercial publishers currently being the most common publisher of OA articles. The category of professional non-commercial publishers is a new type of publisher that has rapidly emerged during the last few years, largely attributed to the journals published by the Public Library of Science.

**Figure 5:**
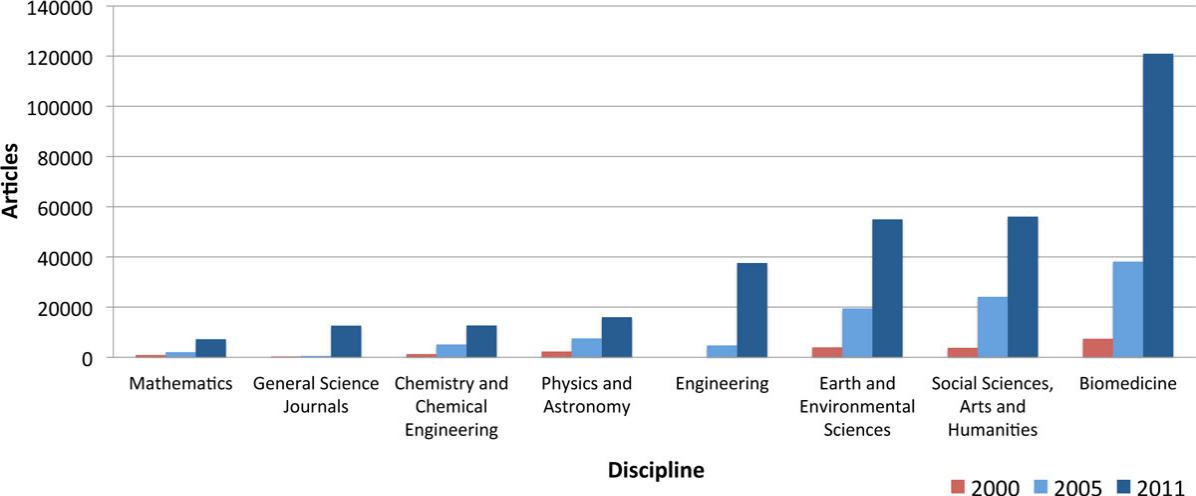
**Open access across major scientific disciplines**.

Figure 5 presents the OA article volumes for the years 2000, 2005 and 2011 split across the major scientific disciplines, with an additional category for general science journals. Throughout the decade, articles in journals broadly related to biomedicine have held the lead in terms of article volume, and since 2005 the gap to the other disciplines has been further extended. Biomedical journals published 120,900 articles in 2011, constituting 35.5% of the total OA article output for the year. In second place in terms of volume for 2011 is the social sciences and humanities, almost tied with earth and environmental sciences in third place, publishing 56,000 and 54,900 articles respectively. Coming in fourth place in terms of size is engineering, which is the discipline that has seen the most dramatic relative growth between 2005 and 2011, from publishing only 4,800 articles in 2005 to 37,500 articles in 2011. In fifth place for 2011 is physics and astronomy with 16,000 articles; however, previous studies have shown there to be particularly strong practice and supporting infrastructure for parallel publication within this discipline, potentially lessening the demand for OA journals [21]. Chemistry and chemical engineering is sixth in terms of size with 12,700 articles in 2011, followed by general science journals and mathematics at the tail end with 12,600 and 7,200 articles respectively. The category of general science journals is a relatively new one with only marginal volume until recently. Journals belonging to this category have little or no limitations with regards to research subject or scope. Though it could be argued that *PLOS ONE *is a general science journal, the vast majority of actual articles published so far have been within the scope of biomedicine, thus that specific journal was placed within the biomedicine category for this coarse disciplinary breakdown.

## Discussion

Over the course of the last decade, OA journal publishing has grown universally across diverse types of journal publishers, geographical regions and scientific disciplines. This has resulted in a continuously growing proportion of journal articles being published OA for each year that has passed, with the most recent measurement from this study being 17% when delayed OA articles with a maximum embargo of 12 months are included. However, despite all the studied dimensions showing increases in annual article output over the decade, the results of the study show that growth has not been uniform across the board. OA publishing seems to be in a very dynamic growth phase, with major shifts in the internal composition happening in a relatively short span of time.

A major strength of the study is associated with the labor-intensive manual approach to data collection, where the annual article volumes for each journal included in the sample was registered for the years 2000 to 2011. This approach reduces the risk of using incorrect, skewed or incomplete source data. The methodological transparency should also enable others to produce comparable numbers to follow-up and compare with the measurements provided here. What can be held as a weakness is the reliance on sampling rather than complete population coverage, however, such an approach is not feasible with the indexing tools currently available and manually collecting the data for over 7,000 journals is a very labor-intensive task.

In comparison with existing studies, this is not only the first study to provide comprehensive gold OA measurement for 2010 and 2011, but the results for the earlier years studied are also more accurate and representative of the actual volumes published at the time. The previous directly comparable study suggested that 191,000 articles were published by full immediate OA journals during 2009 [13], whereas this study suggests the volume for the same year to actually be 225,600. The discrepancy in retrospective annual volumes between these two studies, or any other earlier study using data from the DOAJ, is influenced by the time-lag between the time journals actually start publishing OA and the time they get registered to the DOAJ. In part, this is because journals have to submit a request to the DOAJ to be added, meaning that journals rarely are registered from the first issue they publish, if at all. Another issue is the time the DOAJ takes to process new addition requests; as of September 2012 the backlog of journals currently in queue for evaluation is described as being 'huge' on the DOAJ contact page [20]. Exploring this issue more closely through the sampled journals, it appears that over half of the sampled journals added to the DOAJ during 2010 and 2011 had been publishing OA already prior to 2010, with a handful of cases publishing OA for over a decade prior to DOAJ registration. As was noted in the introduction, most other earlier studies have been limited by only looking at specific OA subsets for specific years, and are thus not directly comparable. However, despite this inability to compare our estimates directly with earlier studies because of methodological incompatibilities, all the results nevertheless speak for the notion of a strong longitudinal growth for OA, particularly so for the biomedical research field.

The results, in particular the finding that approximately 17% of scholarly journal articles are already now made openly available on the Web within a year by the publishers, should be an important input for the policy discussions on OA in venues like the US Congress, the European Commission and the UK Finch Committee that recently published its report with OA-guidelines for British research funders [6]. This study also sheds new light on the relative contributions of the two complementary routes for achieving OA, the publisher-provided gold route and the author-provided green route, indicating that the contribution of gold (both immediate and articles withheld for short embargo periods) is much larger than many earlier estimates. The results should also be considered together with two other recent studies [3,9]. These studies suggest that the level of article-processing charges paid is on average around 900 USD, which is lower than generally believed, and that the scientific impact of OA journals founded in the last decade, and in particular in biomedicine, is on par with similar subscription journals, as measured by average number of citations.

It no longer seems to be a question whether OA is a viable alternative to the traditional subscription model for scholarly journal publishing; the question is rather when OA publishing will become the mainstream model. What remains to be seen is whether the growth will continue at a similar rate as measured during last few years, or if it will accelerate to an even steeper part of the S-shaped adoption pattern typical of many innovations [29]. As in many other markets where the Internet has thoroughly rewritten the rules of the game, an interesting question is if new entrants, like Public Library of Science and BioMed Central, will take over the market or if the old established actors, commercial and society publishers with subscription-based revenue models, will be able to adapt their business models and regain the ground they have so far lost. Future studies on the internal structure of OA publishing are likely to witness the anatomy transforming yet again. Most of the major internal shifts in OA journal publishing have only happened fairly recently during the last few years and, judging by the momentum at which things are moving, it is hard to imagine the internal dynamics settling down any time soon.

## Competing interests

The authors declare that they have no competing financial interests. B-CB founded an OA journal in the 1990s and is emeritus Editor-in-Chief. B-CB is a current board member of the Open Access Scholarly Publishers Association.

## Authors' contributions

ML and B-CB conceived, designed and coordinated the study. ML handled most of the data collection and analysis. Both authors participated equally to interpretation of the results and writing of the manuscript. Both authors have read and approved the final manuscript.

## Authors' information

ML is a doctoral student in Information Systems Science at the Hanken School of Economics, Helsinki, Finland. B-CB is professor of Information Systems Science at the Hanken School of Economics, Helsinki, Finland.

## Pre-publication history

The pre-publication history for this paper can be accessed here:

http://www.biomedcentral.com/1741-7015/10/124/prepub
